# ID 216 - Monitoramento do uso de quimioterápicos para tratamento da leucemia mieloide crônica (LMC) no Sistema Único de Saúde (SUS)

**DOI:** 10.5327/2237-9622.2025.v34s1.216

**Published:** 2025-11-25

**Authors:** Amanda Oliveira Lyrio, Annemeri Livinalli, Ana Carolina de Freitas Lopes, Luciene Fontes Schluckebier Bonan

**Keywords:** leucemia mieloide crônica, quimioterápicos, monitoramento, tratamento oncológico

## Abstract

**Introdução:**

A avaliação do tratamento da leucemia mieloide crônica (LMC) no SUS enfrenta desafios devido à falta de estudos detalhados sobre os quimioterápicos utilizados, dificultados pela falta de informações padronizadas. O financiamento oncológico no SUS é baseado na Autorização de Procedimentos Ambulatoriais (Apac), que define o tipo de câncer e a linha de tratamento, mas a escolha do quimioterápico fica a critério dos hospitais. O registro dos medicamentos é feito em campos abertos, preenchidos de forma heterogênea, dificultando a análise sistemática. Em 2022, o Ministério da Saúde introduziu o campo "Medicamentos Antineoplásicos Informados" para aprimorar o monitoramento, porém esses dados não são retroativos. Este estudo visa descrever o uso de quimioterápicos para LMC em segunda e terceira linhas de tratamento no SUS, entre 2019 e 2022.

**Método:**

Estudo descritivo com dados retrospectivos e administrativos de mundo real, abrangendo todo o território nacional. Dados de dispensação foram obtidos da Sala Aberta de Situação de Inteligência em Saúde (Sabeis), que utiliza informações individualizadas e anonimizadas do Sistema de Informações Ambulatoriais do SUS (SIA/SUS), via Apac. Pacientes com registros de quimioterapia para LMC em segundas e terceiras linhas de tratamento foram incluídos, identificados pelos códigos do Sistema de Gerenciamento da Tabela de Procedimentos, Medicamentos e Órteses, Próteses e Materiais Especiais do SUS (Sigtap): 0304030228, 0304030147, 0304030082, 0304030120, 0304030139 e 0304030148. Os quimioterápicos foram agrupados em três categorias: dasatinibe, nilotinibe e outros, conforme os esquemas terapêuticos registrados. Termos com erros ortográficos e nomes comerciais foram incluídos, e os dados foram revisados para garantir consistência com as categorias definidas.

**Resultados:**

Houve aumento geral no uso de quimioterápicos ao longo do período. O nilotinibe teve crescimento de 50% entre 2019 e 2022. O uso de outros quimioterápicos aumentou 49%, indicando diversificação no tratamento da LMC. O dasatinibe apresentou crescimento modesto (6%). Na segunda linha, o dasatinibe foi o mais utilizado até 2021, mas foi superado pelo nilotinibe em 2022, com 45% dos usuários, comparado a 42% do dasatinibe. A categoria de outros quimioterápicos permaneceu estável, com 13% dos pacientes em 2022. Na terceira linha, o número de pacientes foi menor (5.539 na segunda linha e 620 na terceira). Nessa última, outros quimioterápicos foram os mais utilizados durante todo o período, com 43% dos pacientes em 2022, seguidos por nilotinibe (35%). O dasatinibe foi o menos utilizado na terceira linha (22%).

**Conclusão:**

Os resultados mostram que dasatinibe e nilotinibe predominam na segunda linha de tratamento da LMC no SUS, com nilotinibe emergindo como principal escolha em 2022. Na terceira linha, o nilotinibe manteve relevância, mas o uso de outras terapias destaca a diversificação do manejo da LMC. O aumento no uso de diferentes quimioterápicos reforça a necessidade de monitoramento contínuo e padronizado dos medicamentos oncológicos no SUS, essenciais para o desenvolvimento de políticas públicas eficazes e otimização de recursos.

